# Correction: Polymers as advanced antibacterial and antibiofilm agents for direct and combination therapies

**DOI:** 10.1039/d3sc90061d

**Published:** 2023-04-05

**Authors:** Zhangyong Si, Wenbin Zhong, Dicky Prananty, Jianghua Li, Chong Hui Koh, En-Tang Kang, Kevin Pethe, Mary B. Chan-Park

**Affiliations:** a School of Chemical and Biomedical Engineering, Nanyang Technological University Singapore 637459 Singapore MBEChan@ntu.edu.sg; b Department of Chemical & Biomolecular Engineering, National University of Singapore 4 Engineering Drive 4, Kent Ridge Singapore 117585 Singapore; c Lee Kong Chian School of Medicine, Nanyang Technological University Singapore 636921 Singapore; d School of Biological Sciences, Nanyang Technological University Singapore 637551 Singapore; e School of Physical & Mathematical Sciences, Nanyang Technological University Singapore 637371 Singapore

## Abstract

Correction for ‘Polymers as advanced antibacterial and antibiofilm agents for direct and combination therapies’ by Zhangyong Si *et al.*, *Chem. Sci.*, 2022, **13**, 345–364, https://doi.org/10.1039/D1SC05835E.

The authors regret that the name of one of the authors (Wenbin Zhong) was shown incorrectly in the original Review Article. The corrected author list is as shown above.

The Royal Society of Chemistry apologises for these errors and any consequent inconvenience to authors and readers.

## Supplementary Material

